# Collaborative robotic biomechanical interactions and gait adjustments in young, non-impaired individuals

**DOI:** 10.1186/s12984-016-0166-1

**Published:** 2016-06-16

**Authors:** Valdeci C. Dionisio, David A. Brown

**Affiliations:** Physical Therapy Course, Federal University of Uberlandia, 1876 Benjamin Constant St, Uberlandia, Minas Gerais Brazil; Department of Physical Therapy, University of Alabama at Birmingham, 1720 2nd Avenue South, Birmingham, AL 35294 USA

**Keywords:** Biomechanics, Gait, Human-robot interaction, Motion analysis, Rehabilitation robots

## Abstract

**Background:**

Collaborative robots are used in rehabilitation and are designed to interact with the client so as to provide the ability to assist walking therapeutically. One such device is the KineAssist which was designed to interact, either in a self-driven mode (SDM) or in an assist mode (AM), with neurologically-impaired individuals while they are walking on a treadmill surface. To understand the level of transparency (i.e., interference with movement due to the mechanical interface) between human and robot, and to estimate and account for changes in the kinetics and kinematics of the gait pattern, we tested the KineAssist under conditions of self-drive and horizontal push assistance. The aims of this study were to compare the joint kinematics, forces and moments during walking at a fixed constant treadmill belt speed and constrained walking cadence, with and without the robotic device (OUT) and to compare the biomechanics of assistive and self-drive modes in the device.

**Method:**

Twenty non-neurologically impaired adults participated in this study. We evaluated biomechanical parameters of walking at a fixed constant treadmill belt speed (1.0 m/s), with and without the robotic device in assistive mode. We also tested the self-drive condition, which enables the user to drive the speed and direction of a treadmill belt. Hip, knee and ankle angular displacements, ground reaction forces, hip, knee and ankle moments, and center of mass displacement were compared “in” vs “out” of the device. A repeated measures ANOVA test was applied with the three level factor of condition (OUT, AM, and SDM), and each participant was used as its own comparison.

**Results:**

When comparing “in” and “out” of the device, we did not observe any interruptions and/or reversals of direction of the basic gait pattern trajectory, but there was increased ankle and hip angular excursions, vertical ground reaction force and hip moments and reduced center of mass displacement during the “in device” condition. Comparing assistive vs self-drive mode in device, participants had greater flexed posture and accentuated hip moments and propulsive force, but reduced braking force.

**Conclusions:**

Although the magnitudes and/or range of certain gait pattern components were altered by the device, we did not observe any interruption from the mechanical interface upon the advancement of the trajectories nor reversals in direction of movement which suggests that the KineAssist permits relative transparency (i.e.. lack of interference of movement by the device mechanism) to the individual’s gait pattern. However, there are interactive forces to take into account, which appear to be overcome by kinematic and kinetic adjustments.

## Background

A common problem in individuals with neurologic disorders is gait impairment. In general, ability to recover walking ability is one of the major goals of rehabilitation [1], and interactive robotic devices are becoming more common as one approach to restoring walking ability [2, 3]. New robotic device designs targeting gait and balance recovery have undergone many advances in the past few years. In many cases, the interface is designed to interact with the client so that it can provide the ability to assist or resist walking in a therapeutic manner [2–5]. Nevertheless, one of the major features to include is components that encourage active initiation and engagement of walking during training, which is essential for improvement with motor learning [6]. Thus, well-designed, relatively transparent (i. e., minimal interference with movement due to interaction with the device mechanism) are essential to assure that movement is not arrested or the direction of trajectories are reversed so that the robotic interfaces have potential to improve a patients’ ability and to better stimulate essential nervous system activity for motor learning.

Collaborative robots, or “cobots”, are devices that act as assistants to the human operator. These robots are intrinsically passive, but they are able to assist a movement from action of an operator, using a sophisticated technology developed for haptic sensing [7, 8]. These characteristics are important to reduce the interaction forces between robot and user and increase the stability and human precision of the movement [7].

The KineAssist is a new human-machine collaborative robotic device which has a pelvic harness system embedded in a relatively transparent mechanism that is attached to the hip/pelvis that senses fore-aft horizontal intention forces that drive a treadmill. Treadmill surface movement speed is proportional to the applied force, and provides a safe environment against falls for individuals with balance impairment [9, 10]. Peshkin et al. [11] described design details of the KineAssist and they reported that the trunk and pelvis mechanism were designed to allow patients’ natural walking, allowing full degrees of freedom, however walking speed was slowed while in the device, indicating that interactive forces between the patient and the device exact some level of resistance to be overcome.

The KineAssist is now being marketed and sold to rehabilitation clinics that focus on neurologically-impaired individuals who are regaining walking and balance ability. Due to the device’s mechanism that requires self-initiation to drive the response of the treadmill belt surface, clients are engaged in therapeutic regimens that attempt to improve their ability to initiate stepping and balance responses in a safe manner. This self-drive mode characteristic comes potentially at a biomechanical cost, and this study examines the precise nature of that mechanical interaction. In addition, the device can be used in a therapeutic mode that provides assistive forces in the horizontal direction at the pelvis in order to allow individuals to achieve higher walking training speeds without the necessity for the individual to generate greater propulsive forces. The precise nature of how the system achieves the horizontal force assistance is reported in this study, as well.

It is important to characterize the precise human-machine interactions to understand the level of transparency that is produced and to estimate and account for changes in the kinetics and kinematics of the gait pattern as the device is being used therapeutically. The device can be used in two different modes: 1) Assistive Mode (AM) which provides a horizontal “push” that assists with forward directed force generation to the user while walking, and 2) Self-Drive Mode (SDM) which enables the user to drive the speed and direction (forward vs backward) of the treadmill belt while exerting horizontal force, isometrically, against the pelvic mechanism. The mechanism senses the amount of horizontal force generated by the user and uses this signal to drive the motor of the treadmill belt at a speed within a defined force versus velocity relationship. However, currently there are no studies testing the characteristics of this new device when used in assist mode and self-drive mode. For example, the forces imposed by the pelvic system, especially when one or both feet are in contact with the treadmill surface, could modify the generation of the joint moments across the lower limb and might cause interruptions and/or direction reversals in movement trajectory. In order to characterize the user-machine interactions during each of the two modes, we performed an experiment with non-impaired individuals of the therapeutic device that is now being marketed and sold worldwide (www.kineassist.com). The first aim of this study was to compare the joint kinematics, forces and moments during walking at a fixed constant treadmill belt speed and constrained walking cadence, with and without the robotic device. Since the AM condition uses a fixed treadmill belt speed, the comparisons were between AM and OUT conditions. We hypothesized that the relative transparency of the robotic system will allow for similar kinematic trajectories (i. e. natural walking) without interruption and/or direction reversals, however, due to external forces exerted by the device on the user in the forward propulsive direction, we expected changes to ground reaction forces, and joint moments “in” versus “out” of the device during assistive mode (OUT and AM conditions). The second aim was to compare the biomechanics of assistive (AM) and self-drive (SDM) modes in the device so that we could determine the relative mechanical impact of user-generated forces, sensed by the pelvic mechanism, that drive the treadmill belt at proportional speeds. With this aim we hypothesized that, when the person attempts to control a target speed in SDM, we would observe adjustments in kinematics and kinetics in response to interactions with the pelvic mechanism in order to perform the task compared to the AM condition.

## Methods

### Participants

Twenty non-neurologically impaired individuals participated in this study (10 male and 10 female) between 19–35 years old (23.8 ± 3.62 years). The criteria for recruitment were that the participants have no history of neurologic and/or musculoskeletal disorders which can affect the lower limb, walking and/or balance. Also, candidates were excluded if they presented with cardiac arrhythmia, hypertension, or any known gait abnormality such as lower limb pain that would bias the results of this study. Informed consent was obtained from all subjects with the approval of the University of Alabama at Birmingham Institutional Review Board for Human Research (protocol X150417006). Table 1 shows the characteristics of participants.

**Table 1:** Characteristics of participants

Participants	Mean (DP)
Age (years)	23.8 (3.62)
Height (m)	1.69 (0.09)
Weight (Kg)	69.99 (12.45)
Gender	Male (10); Female (10)
IPAC activity level	High (9); Moderate (11)

### Instrumentation

The KineAssist Gait and Balance Training System™ (KineAssist, HDT Global, Solon OH) was tethered to a Bertec force plate instrumented treadmill (Bertec Corporation, Columbus, OH, USA). The treadmill’s contact surface measures 1.75 × 1 m, with two separate force plates used to record the ground reaction forces (Fx, Fy and Fz) and moments (Mx My and Mz) in all directions with sampling rate of 1000 Hz. The KineAssist was described in details in previous studies [11–13]. Assist Mode (AM) is generated by the pelvis mechanism applying force the individual’s pelvis/hip at the approximate center of mass while the treadmill belts are driven to move at a fixed speed, thereby creating a situation where the device is essentially “pushing” the individual to virtually move forward. Since the pelvis mechanism does not allow forward or backward movement by the hip/pelvis of the individual, the lower limbs that are in contact with the treadmill surface are assisted in moving backward with the fixed movement of the treadmill belt at 1.0 m/s. Self-Drive Mode (SDM) is generated by horizontal forces applied by the user to the pelvic mechanism in order to drive the speed and direction (forward vs backward) of the treadmill belt. The harness system has sensors that are integrated with a servomechanism that uses the measured horizontal force signal to dictate the treadmill belt speed based on a predictable linear relationship controlled by software [10]. This software is able to control the force/velocity relationship (damping) and resistance (deadband) to move the belts. The damping is related to sensitivity (i.e. slope of the relationship between force and velocity) to move the treadmill belts, while the deadband is related to minimum force required to initiate motion of the treadmill belt (i.e. y-intercept of the force vs velocity relationship) [10].

During both AM and SDM, participants were asked to walk at a constant speed, with a fixed cadence using a metronome to provide a cue, and to assure that the overall spatiotemporal variables of walking (velocity, cadence, and average step length) were comparable during the various repeated measure conditions. During SDM, walking involved supplying visual feedback about target speed with an oscilloscope (TDS1012, Tektronix, Beaverton, OR) that displayed instantaneous treadmill belt walking speed supplied by an in-house built velocity meter, and participants were asked to maintain walking speed at an average of 1.0 m/s.

**Fig. 1:**
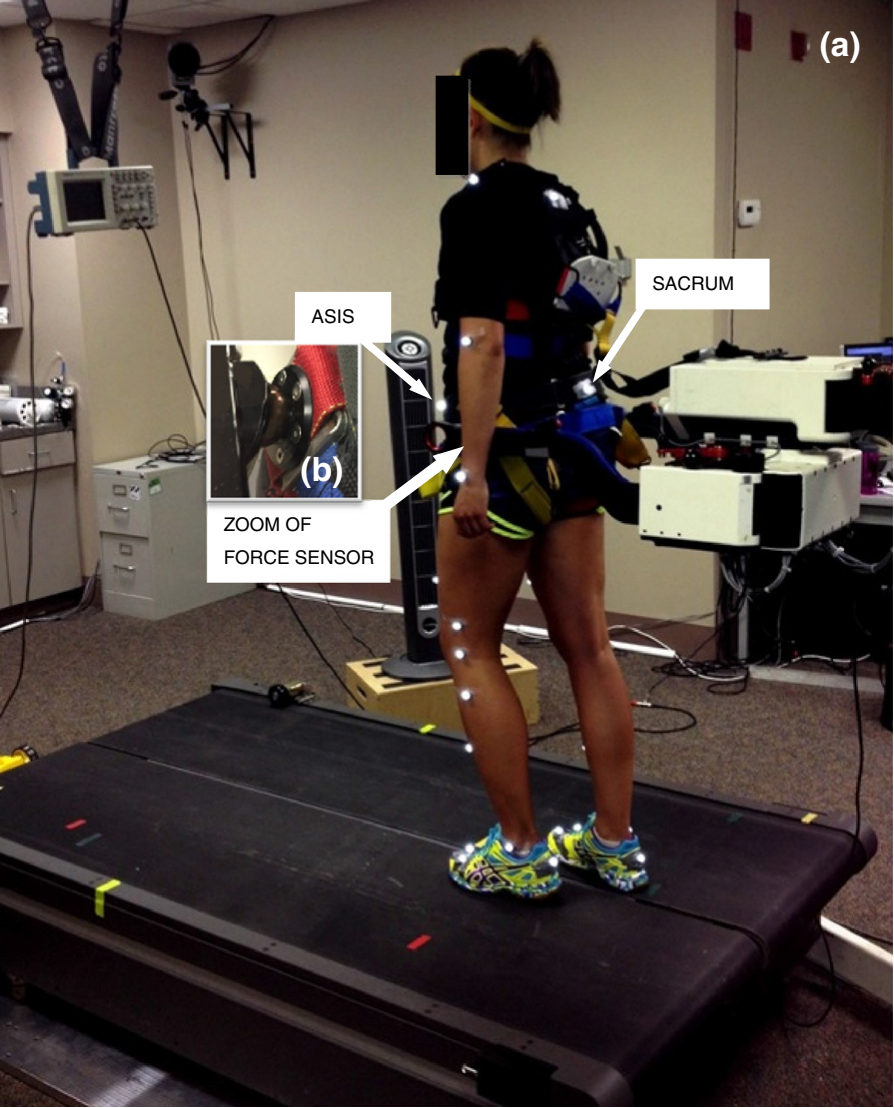
Experimental setup (**a**). Note the markers over anterior superior iliac spines (ASIS) and sacrum over straps, and (**b**) a close-up view of the force sensor localized on the pelvic mechanism

Thirty-three reflective markers were placed on the arms, legs, feet and trunk, and twelve rigid body segments standard were created to biomechanical calculations using Visual3D (C-Motion, Germantown, MD, USA) and one more marker was placed on each arm of robot (total 35 markers). At the pelvis, the markers were placed on anterior superior iliac spines and sacrum over straps (Fig. 1a). Positions of the markers were captured by eight high-speed digital cameras that operated at a 100 Hz sampling frequency by Qualisys Track Manger Software (version 2.8) which synchronized with the Bertec force plates.

### Experimental procedures

After providing consent, participants were invited to fill in the short version of the International Physical Activity Questionnaire (IPAQ) in order to verify the participant’s physical activity level. Participant weight, height, age, and sex were also recorded. As a precaution, blood pressure and heart rate were measured and then markers were placed on the participant’s body.

Participants were asked to walk on the treadmill during 30 s in each of three conditions, outside KineAssist with the treadmill set to a fixed speed of 1.0 m/s (OUT) and attached to KineAssist in both the assistive (AM) and self-drive (SDM) modes. The AM condition was performed with the participant walking at a set treadmill speed of 1.0 m/s, but while prevented from moving to positions either forward or backward on the treadmill belt due to the KineAssist pelvic mechanism constraint. However, side to side and rotation about the vertical and horizontal axis were all free to move. As the person walked at a set speed, the KineAssist generates a forward directed force, thereby assisting with maintaining a given walking speed. In contrast, the SDM condition requires that the participant generate a net propulsive force against the pelvic mechanism so that the force sensors embedded in the harness system (Fig. 1b) can generate a command signal that drives the treadmill belt at a speed that is proportional to the applied force. The force/velocity relationship (damping) in this mode was set to 80 N*s/m and the resistance (deadband) was 10 N to initiate treadmill movement.

To provide a short familiarization to the KineAssist device, the participants first walked on the treadmill using the KineAssist and were asked to practice maintaining cadence and speed using the AM and SDM modes. During this period, we calculated the appropriate cadence for comfortable walking at speed 1.0 m/s in AM. The same cadence metronome feedback was used for all conditions (OUT, AM and SDM). The data were synchronized and recorded for analysis.

### Data processing

Data were post-processed using Visual3D (C-Motion, Germantown, MD, USA) to calculate the relative joint angles and joint moments (ankle, hip and knee) from kinematic and force plate data using an inverse dynamic routine. The anthropometric model based in Helen Hayes pelvis segments was applied to calculate hip joint center and, the knee and ankle joints center was calculated from markers placed in lateral and medial on each joint. Each segment received three markers to build the model. The model was applied on static data and then applied on dynamic data. Participant’s anthropometric data for each trial was applied to the model. The joint angles and joint moments (ankle, hip and knee) also were calculated from kinematic and force plate data following the instructions in Visual3D. After calculation, the joint angles and moments signals were filtered using a Butterworth filter at 6 Hz. Then, data from KineAssist (anterior-posterior and vertical forces) and from Visual 3D were exported to MatLab® for post-processing (Matworks®, version R2014b, 8.4.0.150421), which involved filtering of ground reaction forces using a Butterworth filter at 10 Hz, calculation of heel strikes and toe off, to normalize by gait cycle. We calculated the average, maximum, and minimum values for all variables, angular excursion of the joints (maximum minus minimum values) and center of mass excursion (maximum minus minimum values) across all steps. The center of mass displacement was calculated in medio-lateral, anterior-posterior and vertical directions. Vertical force was integrated in three sub-phases of the stance phase. The early stance was calculated from heel stride of one leg (reference) until toe off of opposite leg; the mid-stance was calculated from toe off until heel stride of opposite leg; and the late stance was calculated from heel stride of opposite leg until toe off the reference leg. For anterior-posterior force, the impulse was calculated considering the braking (positive values) and propulsive (negative) forces. All forces and joint moments were normalized by body mass. The same procedure was used for all conditions.

### Statistical analysis

Normality of the maximum and minimum values of angles, forces and moments, angular excursion and center of mass displacement were tested using the Shapiro-Wilk test [14]. All of these variables presented with normal distributions. Therefore, we used a repeated measures ANOVA test with the three level factor of condition (OUT, AM, and SDM), and each participant was used as its own comparison. When there was a significant condition effect, the post hoc comparison was used comparing two factors separately [OUT × AM] and [AM x SDM], because we wished to compare the main effects of walking “in” vs “out” of the KineAssist during fixed treadmill speed conditions (OUT vs AM) and, separately, the main effects of walking in the KineAssist under self-drive vs assisted conditions (SDM vs AM). All tests were performed in IBM_©_ SPSS_©_ (version 22.0.0.0) and we used for statistical significance level at 0.05 for all comparisons.

## Results

All participants were able to follow the metronome and use the oscilloscope, performing the tasks in the appropriate way. Detailed results are presented in this section and then presented in a summary fashion with a table at the end of the results section.

### OUT vs assistive mode walking in the device: sagittal plane

**Fig. 2:**
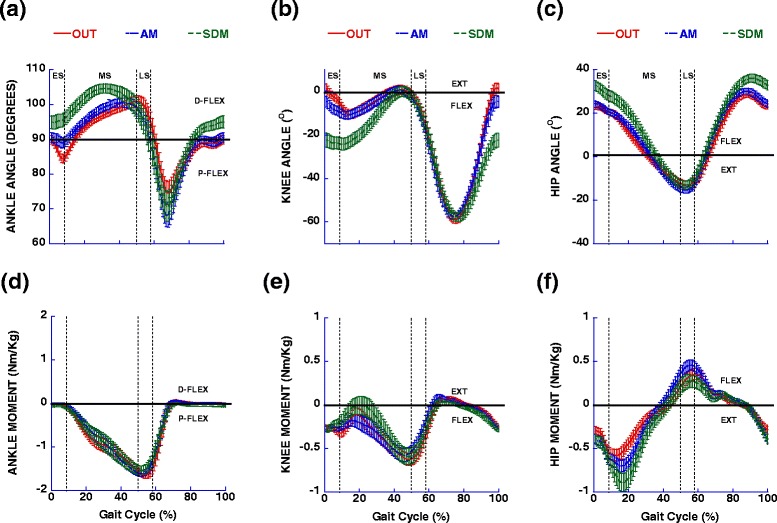
Mean and standard deviation for ankle, knee and hip angles (**a**-**c** respectively) and moments (**d**-**f** respectively) in the sagittal plane for left limb showed during OUT, AM and SDM conditions. Vertical dotted lines represent temporal markers of the various stance phases, ES = early stance, MS = mid-stance and LS = late stance. D-Flex = dorsiflexion, P-Flex = plantar flexion, Flex = Flexion, Ext = extension

**Fig. 3:**
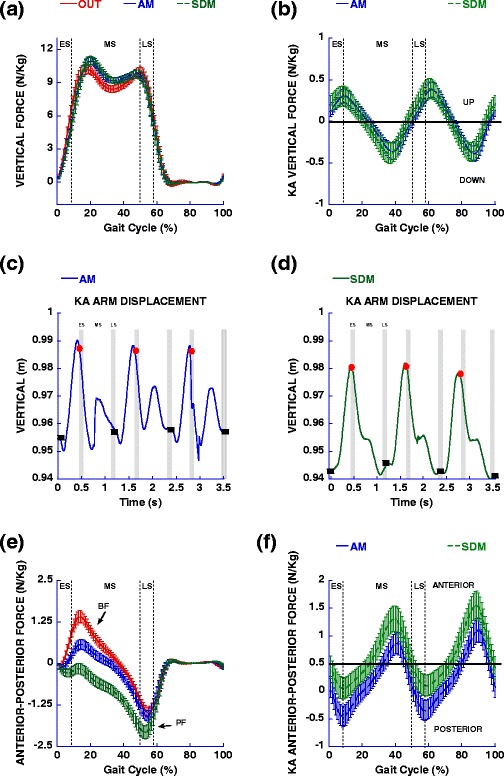
Vertical (**a**) and anterior-posterior (**e**) reaction forces from force plate are showed for left limb during OUT, AM and SDM conditions. Also, vertical (**b**) and anterior-posterior (**f**) forces from the robot-pelvis interface and vertical displacement (temporal series) during AM (**c**) and SDM (**d**) conditions. Vertical dotted lines and crosshatch bars represent temporal markers of the various stance phases, ES = early stance, MS = mid-stance and LS = late stance. The red circle = heel stride and black square = toe off. The arrows show the BF = braking force and PF = propulsive force

The results revealed that, comparing OUT vs AM condition, kinematic trajectories of the walking was very similar between the conditions, and without interruption and/or reversals in direction, but there was greater ankle plantar flexion and hip angular excursion that occurred with the AM condition (Table 2; Fig. 2a, c). With kinetic measures (maximum and minimum values), hip extensor and flexor moments (Fig. 2f) and vertical force (Fig. 3a) were larger, and braking force (Fig. 3e) was reduced in the AM condition, while propulsive force was similar (Table 2). For horizontal impulses calculated for braking and propulsive forces (Fig. 3e), braking impulse was reduced, while the propulsive impulse was increased in AM condition (Table 2).

**Table 2:** Mean and standard deviation showed for kinematic and kinetic in sagittal plane for left limb

	Conditions			Statistical analysis			
Variables	OUT	AM	SDM	OUT vs AM		AM vs SDM	
Sagittal kinematic (degree)				*F*	*p*	*F*	*p*
Maximum ankle (D-FLEX)	79.34 (4.42)	79.69 (4.49)	83.97 (4.04)	0.264	0.614	25.858	**0.000**
Minimum ankle (P-FLEX)	51.14 (6.95)	44.78 (6.32)	45.93 (7.64)	32.797	**0.000**	2.570	0.125
Ankle angular excursion	28.19 (5.61)	34.90 (4.18)	38.03 (6.73)	28.089	**0.000**	7.571	**0.013**
Maximum hip (FLEX)	29.09 (7.33)	30.47 (6.85)	38.33 (7.82)	0.702	0.413	51.824	**0.000**
Hip angular excursion	42.40 (4.71)	46.09 (6.63)	53.60 (7.33)	7.725	**0.012**	28.789	**0.000**
Maximum knee (EXT)	4.35 (4.87)	3.27 (4.51)	0.40 (4.75)	2.019	0.172	9.851	**0.005**
Minimum knee (FLEX)	−59.02 (4.62)	−58.26 (4.34)	−60.81 (4.34)	0.685	0.418	14.982	**0.001**
Sagittal Kinetic (Nm/Kg)							
Maximal anterior-posterior force (BF)	1.44 (0.27)	0.65 (0.39)	0.11 (0.07)	83.223	**0.000**	34.739	**0.000**
Minimal anterior-posterior force (PF)	−1.52 (0.22)	−1.63 (0.26)	−2.12 (0.30)	2.075	0.166	66.126	**0.000**
Maximal vertical force	10.64 (0.69)	11.11 (0.67)	11.19 (0.70)	7.616	**0.012**	0.503	0.487
Minimal vertical force	−0.30 (0.12)	−0.19 (0.10)	−0.21 (0.11)	50.481	**0.000**	1.718	0.206
Maximal hip moment (FLEX)	0.37 (0.12)	0.46 (0.16)	0.33 (0.18)	11.517	**0.003**	17.541	**0.000**
Minimal hip moment (EXT)	−0.61 (0.09)	−0.73 (0.18)	−0.92 (0.31)	12.422	**0.002**	12.885	**0.002**
Sagittal impulse (Ns/kg)				*F*	*p*	*F*	*p*
Breaking force	0.30 (0.05)	0.14 (0.07)	0.03 (0.06)	90.621	**0.000**	16.554	**0.001**
Propulsive force	−0.28 (0.08)	−0.36 (0.13)	−0.64 (0.18)	4.819	**0.041**	37.438	**0.000**
Vertical force ES	0.92 (0.14)	0.82 (0.15)	0.85 (0.12)	6.007	**0.024**	0.863	0.365
Vertical force MS	3.65 (0.41)	3.95 (0.60)	3.81 (0.41)	10.400	**0.004**	2.471	0.132

Statistical analysis includes post hoc comparisons of two factors separately using repeated measures OUT vs AM and AM vs SDM conditions. When repeated measures overall testing showed that the main effects were not significant (*p* > 0.05), post-hoc tests were not conducted and not presented. Bold values indicate significant differences (*p* < 0.05). D-FLEX: dorsiflexion; P-FLEX: plantar flexion; FLEX: flexion; EXT: extension; BF: braking force; PF: propulsive force; ES: early stance; MS: mid-stance; LS: late stance

### OUT vs assistive mode walking in the device: frontal plane

**Fig. 4:**
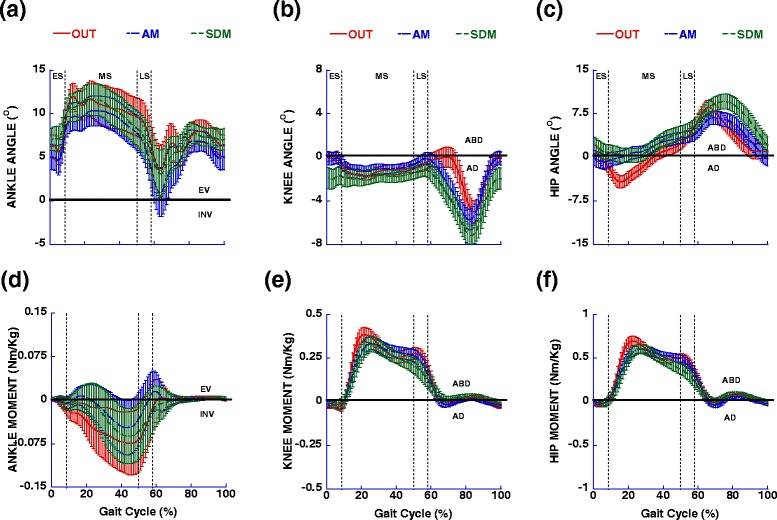
Mean and standard deviation for ankle, knee and hip angles (**a**-**c** respectively) and moments (**d**-**f** respectively) in the frontal plane for left limb showed during OUT, AM and SDM conditions. Vertical dotted lines represent temporal markers of the various stance phases, ES = early stance, MS = mid-stance and LS = late stance. EV = eversion, INV = inversion, ABD = abduction, AD = adduction

The results revealed that comparing OUT vs AM condition the frontal plane kinematic trajectories were very similar between the conditions, except with lesser hip adduction occurring in the AM condition (Fig. 4c; Table 3). The kinetic analysis showed that lateral force and hip moment (Fig. 4f) were reduced in the AM condition (Table 3).

**Table 3:** Mean and standard deviation showed for kinematic and kinetic in frontal plane for left limb

	Conditions			Statistical analysis			
Variables	OUT	AM	SDM	OUT vs AM		AM vs SDM	
Frontal kinematic (degree)				*F*	*p*	*F*	*p*
Maximum hip (ABD)	8.70 (2.96)	6.86 (3.69)	9.80 (4.35)	3.718	0.070	25.587	**0.000**
Minimum hip (AD)	−4.51 (4.29)	−1.43 (4.77)	−1.11 (6.00)	11.413	**0.003**	0.363	0.554
Hip angular excursion	13.58 (3.56)	9.24 (2.46)	12.05 (3.56)	22.946	**0.000**	24.612	**0.000**
Maximum knee (ABD)	2.74 (3.71)	1.88 (4.23)	1.02 (4.40)	2.231	0.152	3.541	0.075
Minimum knee (AD)	−6.22 (4.14)	−7.31 (5.18)	−8.38 (5.78)	1.974	0.176	8.487	**0.009**
Frontal kinetic (Nm/Kg)							
Maximal lateral force	0.90 (0.18)	0.75 (0.21)	0.74 (0.23)	22.410	**0.000**	0.064	0.802

Statistical analysis includes post hoc comparisons of two factors separately using repeated measures OUT vs AM and AM vs SDM conditions. When repeated measures overall testing showed that the main effects were not significant (*p* > 0.05), post-hoc tests were not conducted and not presented. Bold values indicates significant differences (*p* < 0.05). ABD: abduction; AD: adduction

### OUT vs assistive mode walking in the device: center of mass displacement

**Fig. 5:**
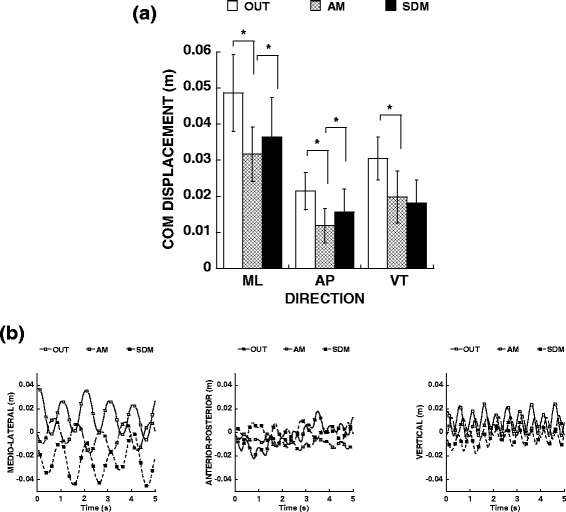
Mean and standard deviation for center of mass (COM) displacement for a representative participant in (**a**) medio-lateral (ML), anterior-posterior (AP) and vertical (VT) directions showed during OUT, AM and SDM conditions. In (**b**) the temporal series (5 s) of COM displacement showed in ML, AP and VT directions from one representative participant. * *p* < 0.05

Analysis of center of mass displacement reveled that comparing OUT vs AM conditions, center of mass displacement was reduced in medio-lateral (*F* = 101.784, *p* = 0.000), anterior-posterior (*F* = 40.450, *p* = 0.000) and vertical (*F* = 48.190, *p* = 0.000) directions in the AM condition (Fig. 5a).

### Assistive mode vs self-drive mode walking in the device: sagittal plane

The participants were able to follow the instructions and successfully maintain the speed close to the displayed target of 1.0 m/s (mean of 0.99 ± 0.02 m/s) in self-drive condition. Comparing the AM vs SDM condition, there was larger ankle and hip angular excursion in the SDM condition, while the knee excursions were similar (Table 2). However, during early stance the knee was more flexed and then moved into extension during mid-stance (Fig. 2b). As a consequence, knee angular excursions and maximal and minimal values were similar over the course of the entire stance phase. With kinetics, vertical force (Fig. 3a) was similar between conditions, but hip extensor moment (Fig. 2f), and propulsive force were larger and braking force (Fig. 3e) and hip flexor were reduced in the SDM condition (Table 2).

### Assistive mode vs self-drive mode walking in the device: frontal plane

There was larger hip abduction (Fig. 4c) and the knee was more adducted (Fig. 4b) in the SDM condition, but the ankle was similar between conditions (Table 3). For kinetic measures, there was no difference across the conditions.

### Assistive mode vs self-drive mode walking in the device: center of mass displacement

Center of mass displacements in medio-lateral (*F* = 5.305, *p* = 0.033) and anterior-posterior (*F* = 9.656, *p* = 0.006) directions were increased in the SDM condition, however, in vertical (*F* = 1.082, *p* = 0.311) was similar between conditions (Fig. 5a).

Due to the complex nature of the changes in kinetic and kinematic parameters, we have provided an overall summary of results in Table 4.

**Table 4:** This table shows the summary of statistically significant differences for all comparisons in sagittal and frontal planes

Variables	OUT vs AM condition		AM vs SDM condition	
	Sagittal plane	Frontal plane	Sagittal plane	Frontal plane
Angular displacement	• Ankle plantar flexion larger in AM • Hip angular excursion larger in AM	• Hip adduction and angular excursion smaller in AM	• Ankle dorsiflexion and angular excursion larger in SDM • Hip flexion and angular excursion larger in SDM • Knee flexion larger and extension smaller in SDM	• Hip abduction and angular excursion larger in SDM • Knee adduction larger in SDM
Forces	• Vertical force larger in AM • Braking force smaller in AM	• Lateral force smaller in AM	• Braking force smaller, but propulsive force larger in SDM	
Moments	• Hip extensor and flexor moments larger in AM	• Hip abduction moment smaller in AM	• Hip flexor moment smaller, but hip extensor moment larger in SDM	
Impulses	• Braking impulse smaller and propulsive impulse larger in AM • Vertical impulse smaller during early stance, but larger during mid-stance in AM		• Braking impulse smaller, but propulsive impulse larger in SDM	
Center of mass displacement	• Center of mass displacement smaller for medio-lateral, anterior-posterior and vertical directions in AM		• Center of mass displacement larger for medio-lateral and anterior-posterior directions in SDM	

## Discussion

### OUT vs AM conditions

The first aim of this study was to compare the joint kinematics, forces and moments during walking at a fixed constant treadmill belt speed and constrained walking cadence, with and without the robotic device. We hypothesized that the relative transparency of the robotic system would allow for similar kinematic trajectories, without interruption and/or direction reversals, however, due to external forces exerted by the device on the user in the forward propulsive direction, we expected changes to ground reaction forces, and joint moments “in” versus “out” of the device during assistive mode (OUT and AM conditions). The results partially supported our hypothesis, since the phasic nature of the kinematic characteristics of walking (i. e. support and swing phases) was similar when comparing OUT and AM conditions in sagittal and frontal planes (Figs. 2a, c and 4a, c). These results confirmed the observations with functional activities in this device [12, 15]. However, in the AM condition there was more angular displacement for the ankle and hip joints in sagittal plane. There were also important kinematic magnitude differences during mid-stance in the sagittal plane. In the AM condition, the trajectory of the pelvic mechanism was in an inferior direction after heel strike and we found the lowest value in mid-stance, about an average of 0.04 m (Fig. 3c). However, the vertical force indicated an upward direction (Fig. 3b). This is because the belts are attached to the participant, and the lower displacement of the pelvic mechanism produced tension in the belt indicating a force amplitude direction in the upper direction. So, the actuated movement of the pelvic mechanism in the vertical direction appears to have pushed the individuals downward against the treadmill, increasing the vertical force (maximum values in mid-stance), and affecting the vertical impulse (Table 3; Fig. 3a). The pelvic mechanism also purposefully limited the anterior displacement of the body (so that interaction forces can be picked up the force sensor in an isometric position), generating a force in posterior direction (Fig. 3f) between early stance and mid-stance sub-phases. The fixed position of the pelvis in the anterior-posterior direction imposed reduction of the braking force, which was reflected in reduced braking impulse (Table 3; Fig. 3e). On the other hand, the flexor and extensor hip moments were increased, presumably to provide stability and acceleration of center of mass in the forward direction. Another interesting aspect is that the knee moment in both conditions was flexor during all of the stance phase (Fig. 2e). The possible explanation is that, over the treadmill, the braking force was slight reduced, especially with the slower speed used in this study (below the comfortable speed) [16]. The reduced braking force and reduced knee moment during walking on the treadmill at a comfortable speed was also observed in a previous study [17]. In the frontal plane, during the AM condition, maximum values for lateral force and hip moment were reduced (Fig. 4e). Taking everything together, the main interactions occurred during mid-stance, where braking force was reduced, but vertical force and hip extensor moment were increased. The movement of the pelvic mechanism in the vertical direction and the reaction force from the pelvic harness system reduced the braking and lateral forces. As a result, the harness system reduced the center of mass displacement (Fig. 5a). Our results are consistent with a previous study, where we observed a reduction of the braking force by horizontal force that was applied close to center of mass, and it was proportional to the resistance that was applied [18]. The participants’ response to forces from the pelvic mechanism served to increase the extensor and flexor hip moments. The simple instruction to the participant to increase the push off provoked the increased hip moment and angular displacement [19]. In this study, the resistance imposed by pelvic mechanism also effectively generated larger hip moment and angular displacement.

### AM vs SDM conditions

The second aim of this study was compare AM and SDM conditions in device and determine the relative impact of user-generated forces, sensed by the pelvic mechanism, that drive the treadmill. We hypothesized that, when the person attempted to control a target speed, they would need to adjust kinematics and kinetics in response to interacting with the pelvic mechanism in order to perform the task compared to the AM condition. The results confirmed our hypothesis, since the results for the sagittal plane showed that, in the SDM condition ankle and hip joints had larger angular excursions and the knee showed larger flexion and smaller extension (Fig. 2a, c; Table 2), which represented a more flexed posture in the SDM condition. Vertical force (Fig. 3a) was not affected, but there was reduced braking force and increased propulsive force in the SDM condition (Fig. 3e). Similarly, the extensor hip moment was increased for the SDM condition compared to the AM condition (Fig. 2f). This situation was reflected by impulse measures, which were reduced for braking impulse, but larger for propulsive impulse (Table 3; Fig. 3e). For the frontal plane, the results showed that larger hip angular excursions appeared with increased hip abduction in the SDM condition (Fig. 4c).

The main results for this comparison occurred during mid-stance and late stance. The challenge imposed by the SDM condition lead to a more flexed posture compared with the AM condition. In addition, the participants had to apply the appropriate force over the sensor embedded in harness system, to drive the treadmill belts at a particular speed. The pelvic mechanism also generated a force in the posterior direction (Fig. 3f) between early stance and mid-stance sub-phases and the inferior trajectory of the mechanism (Fig. 3d), showed a reduced braking force [18], increased extensor hip moment [19] during mid-stance (Fig. 2f) and propulsive force during late stance (Fig. 2c). The propulsive force increase was observed in a previous study and tended to be proportional to the horizontal resistance applied during walking [18]. So, the participants of this study in SDM condition had additional adjustments compared to AM condition.

### Overall human-machine interaction for collaborative robots

While the goal of a robotic walking device is to assist with movement without interfering with the basic mechanics of walking, the nature of the human-machine interface is very difficult to overcome in practice. There are a number of recently developed robot devices applied to improve gait, and in general, all devices have limitations like movements constrained to one anatomical plane (sagittal) which prevents meaningful balance training, reduced degrees freedom on pelvis and or trunk, where the patient is guided during movement [6]. Even with guided movement, non-impaired individuals showed altered angular displacement in lower limb [20, 21] and trunk [22] tested with and without others gait orthosis devices, sometimes increasing [20] and sometimes reducing [21, 22] the movement. In the case of the KineAssist applied over a treadmill, the basic phasic trajectory of the kinetic and kinematic variables were intact (i. e. natural walking) and without arresting or reversing movement trajectories, however important interactive forces were imposed. Despite these interactions, the center of mass displacement had a consistent trajectory (Fig. 5b) and participants were able to respond to interactive forces with mostly small adjustments. Perhaps, these adjustments could serve to increase the rate of learning, at least temporarily, during the execution of a task [23].

The results from this study highlight some important advantages and disadvantages associated with a collaborative robotic system such as the KineAssist. In terms of advantages, the system allows the user to drive the movement of the treadmill belt in SDM which encourages active engagement in the task. Also, while not studied here, the device allows safety and confidence for people with poor balance who are regaining walking ability, since the device will catch a person when their pelvis height drops below a set height. With AM, individuals with reduced force generation during propulsion are afforded opportunities to walk at faster speeds. In terms of disadvantages, the device will reduce comfortable walking speed by as much as 50 % [15] and will require greater mechanical work to raise the vertical trajectory of the center of mass. These two issues will cause extra fatigue and potential muscle soreness when a person exercises with the device. The most obvious disadvantage comes from the caution that any researcher and/or clinician must use when interpreting the trajectory characteristics of the gait pattern as the results of walking in this device should not be used to diagnosis specific gait pattern deficits in individuals with impaired walking.

### Limitations

There are some limitations to be considered in this study. First, the experiment was conducted at one fixed speed which may or may not reflect the person’s comfortable walking speed. However, the speed of 1.0 m/s that we used in this experiment is slower, but close to reported average comfortable walking speeds [16]. Second, we tested at certain damping and deadband settings, and the results could be different with alternatives levels of damping and deadband. A lesser deadband level may have resulted in less effort to drive the treadmill during the SDM condition, while lesser damping settings may have provided greater velocity-dependent sensitivity. However, we selected the particular settings used in this study in order to permit the optimal control of treadmill steadiness and stability. Third, the device used in this study was an older prototype version of the system and has a more massive pelvic mechanism than more current systems that are now on the market. Finally, the participants of this study were healthy and young, classified with moderate to high level of physical activity (Table 1), which provided ideal candidates for adjusting their gait characteristics in the device. Adjustments observed here could be modified in different impaired or older populations but these hypotheses need to be tested with future investigations.

## Conclusions

According to the conditions tested, the results suggested that the collaborative robotic device tested here (The KineAssist) permits relative transparency to an individual’s gait pattern, since there was no interruption nor direction reversal of movement trajectories, but there were important interactive forces which appeared to be present and required adjustment to be overcome by generating kinematic and kinetic adjustments. One must always take into account the human-machine interactions that occur when a person is asked to move while connected with a robotic system.

## Abbreviations

ABD, abduction; AD, adduction; AE, angular excursion; AM, assistive mode; AP, anterior-posterior; ASIS, anterior superior iliac spines; BF, braking force; COM, center of mass; D-FLEX, dorsiflexion; ES, early stance; EV, eversion; EXT, extension; FLEX, flexion; INV, inversion; LS, late stance; ML, medio-lateral; MS, mid-stance; OUT, outside KineAssist; PF, propulsive force; P-FLEX, plantar flexion; SDM, self-drive mode; VT, vertical
